# Supplementary material for: The Cumulative Effect of Expanding the Breadth and Scope of Coverage for Substance Use Disorder Treatment on Behavioral Health Acute Inpatient Admissions: Evidence from Virginia Medicaid
Source: Int J Environ Res Public Health. 2024 Jun 14;21(6):777. doi: 10.3390/ijerph21060777 (PMC11204056; doi:10.3390/ijerph21060777)
Supplement: Supplementary file 1 [file ijerph-21-00777-s001.zip › ijerph-3038732-supplementary.pdf]

Supplementary Materials

Table S1. Comparison of Virginia and North Carolina on behavioral health status, healthcare access and demographics [a].

Behavioral Health Status	Virginia	North Carolina
Prevalence of Mental illness	17.4%	18.8%
Substance use disorders in the past year	7.3%	7.0%
Thoughts of suicide	4.2%	4.6%
**Healthcare Access**	**Virginia**	**North Carolina**
Uninsured	13.5%	13.8%
AMI not received treatment	54.5%	56.5%
AMI reporting unmet need	28.6%	26.5%
Disability who cannot see a doctor due to cost	34.2%	35.2%
**Demographics**	**Virginia**	**North Carolina**
Population census April, 2010	8,631,393	10,439,388
Female	50.8%	51.4%
White	69.4%	70.6%
Black or African American	19.9%	22.2%
High school graduate or higher (>25 years old)	89.7%	87.8%
Median household income (in 2019 dollars)	$74,222	$54,602

[a] 2018 data.

Table S2. Behavioral health disorders ICD-10 codes.

Behavioral Health Conditions	ICD-10 Codes
Adjustment disorders	F4320, F4321, F4322, F4323, F4324, F4325, F4329, F438, F439
Anxiety disorders	F064, F4000, F4001, F4002, F4010, F4011, F40210, F40218, F40220, F40228, F40230, F40231, F40232, F40233, F40240, F40241, F40242, F40243, F40248, F40290, F40291, F40298, F408, F409, F410, F411, F413, F418, F419, F42, F422, F423, F424, F428, F429, F430, F4310, F4311, F4312, F488, F489, R452, R453, R454, R455, R456, R457, R4581, R4582, R4583, R4584
Attention-deficit conduct and disruptive behavior disorders	F900, F901, F902, F908, F909, F910, F911, F912, F913, F918, F919, R460, R461, R462, R463, R464, R465, R466, R467, R4681, R4689
Impulse control disorders NEC	F630, F631, F632, F633, F6381, F6389, F639, R45850
Mood disorders	F0630, F0631, F0632, F0633, F0634, F3010, F3011, F3012, F3013, F302, F303, F304, F308, F309, F310, F3110, F3111, F3112, F3113, F312, F3130, F3131, F3132, F314, F315, F3160, F3161, F3162, F3163, F3164, F3170, F3171, F3172, F3173, F3174, F3175, F3176, F3177, F3178, F3181, F3189, F319, F320, F321, F322, F323, F324, F325, F328, F3281, F3289, F329, F330, F331, F332, F333, F3340, F3341, F3342, F338, F339, F340, F341, F348, F3481, F3489, F349, F39, R4586
Personality disorders	F600, F601, F602, F603, F604, F605, F606, F607, F6081, F6089, F609, F69
Schizophrenia and other psychotic disorders	F060, F062, F200, F201, F202, F203, F205, F2081, F2089, F209, F21, F22, F23, F24, F250, F251, F258, F259, F28, F29
Suicide and intentional self-inflicted injury	R45851, T1491, T1491XA, T1491XD, T1491XS, T360X2A, T360X2D, T360X2S, T361X2A, T361X2D, T361X2S, T362X2A, T362X2D, T362X2S, T363X2A, T363X2D, T363X2S, T364X2A, T364X2D, T364X2S, T365X2A, T365X2D, T365X2S, T366X2A, T366X2D, T366X2S, T367X2A, T367X2D, T367X2S, T368X2A, T368X2D, T368X2S, T3692XA, T3692XD, T3692XS, T370X2A, T370X2D, T370X2S, T371X2A, T371X2D, T371X2S, T372X2A, T372X2D, T372X2S, T373X2A, T373X2D, T373X2S, T374X2A, T374X2D, T374X2S, T375X2A, T375X2D, T375X2S, T378X2A, T378X2D, T378X2S, T3792XA, T3792XD, T3792XS, T380X2A, T380X2D, T380X2S, T381X2A, T381X2D, T381X2S, T382X2A, T382X2D, T382X2S, T383X2A, T383X2D, T383X2S, T384X2A, T384X2D, T384X2S, T385X2A, T385X2D, T385X2S, T386X2A, T386X2D, T386X2S, T387X2A, T387X2D, T387X2S, T38802A, T38802D, T38802S, T38812A, T38812D, T38812S, T38892A, T38892D, T38892S, T38902A, T38902D, T38902S, T38992A, T38992D, T38992S, T39012A, T39012D, T39012S, T39092A, T39092D, T39092S, T391X2A, T391X2D, T391X2S, T392X2A, T392X2D, T392X2S, T39312A, T39312D,

	T39312S, T39392A, T39392D, T39392S, T394X2A, T394X2D, T394X2S, T398X2A, T398X2D, T398X2S, T3992XA, T3992XD, T3992XS, T400X2A, T400X2D, T400X2S, T401X2A, T401X2D, T401X2S, T402X2A, T402X2D, T402X2S, T403X2A, T403X2D, T403X2S, T404X2A, T404X2D, T404X2S, T405X2A, T405X2D, T405X2S, T40602A, T40602D, T40602S, T40692A, T40692D, T40692S, T407X2A, T407X2D, T407X2S, T408X2A, T408X2D, T408X2S, T40902A, T40902D, T40902S, T40992A, T40992D, T40992S, T410X2A, T410X2D, T410X2S, T411X2A, T411X2D, T411X2S, T41202A, T41202D, T41202S, T41292A, T41292D, T41292S, T413X2A, T413X2D, T413X2S, T4142XA, T4142XD, T4142XS, T415X2A, T415X2D, T415X2S, T420X2A, T420X2D, T420X2S, T421X2A, T421X2D, T421X2S, T422X2A, T422X2D, T422X2S, T423X2A, T423X2D, T423X2S, T424X2A,T424X2D, T424X2S, T425X2A, T425X2D, T425X2S, T426X2A, T426X2D, T426X2S, T4272XA, T4272XD, T4272XS, T428X2A, T428X2D, T428X2S, T43012A, T43012D, T43012S, T43022A, T43022D, T43022S, T431X2A, T431X2D, T431X2S, T43202A, T43202D, T43202S, T43212A, T43212D, T43212S, T43222A, T43222D, T43222S, T43292A, T43292D, T43292S, T433X2A, T433X2D, T433X2S, T434X2A, T434X2D, T434X2S, T43502A, T43502D, T43502S, T43592A, T43592D, T43592S, T43602A, T43602D, T43602S, T43612A, T43612D, T43612S, T43622A, T43622D, T43622S, T43632A, T43632D, T43632S, T43692A, T43692D, T43692S, T438X2A, T438X2D, T438X2S, T4392XA, T4392XD, T4392XS, T440X2A, T440X2D, T440X2S, T441X2A, T441X2D, T441X2S, T442X2A, T442X2D, T442X2S, T443X2A, T443X2D, T443X2S, T444X2A, T444X2D, T444X2S, T445X2A, T445X2D, T445X2S, T446X2A, T446X2D, T446X2S, T447X2A, T447X2D, T447X2S, T448X2A, T448X2D, T448X2S, T44902A, T44902D, T44902S, T44992A, T44992D, T44992S, T450X2A, T450X2D, T450X2S, T451X2A, T451X2D, T451X2S, T452X2A, T452X2D, T452X2S, T453X2A, T453X2D, T453X2S, T454X2A, T454X2D, T454X2S, T45512A, T45512D, T45512S, T45522A, T45522D, T45522S, T45602A, T45602D, T45602S, T45612A, T45612D, T45612S, T45622A, T45622D, T45622S, T45692A, T45692D, T45692S, T457X2A, T457X2D, T457X2S, T458X2A, T458X2D, T458X2S, T4592XA, T4592XD, T4592XS, T460X2A, T460X2D, T460X2S, T461X2A, T461X2D, T461X2S, T462X2A, T462X2D, T462X2S, T463X2A, T463X2D, T463X2S, T464X2A, T464X2D, T464X2S, T465X2A, T465X2D, T465X2S, T466X2A, T466X2D, T466X2S, T467X2A, T467X2D, T467X2S, T468X2A, T468X2D, T468X2S, T46902A, T46902D, T46902S, T46992A, T46992D, T46992S, T470X2A, T470X2D, T470X2S, T471X2A, T471X2D, T471X2S, T472X2A, T472X2D, T472X2S, T473X2A, T473X2D, T473X2S, T474X2A, T474X2D, T474X2S, T475X2A, T475X2D, T475X2S, T476X2A, T476X2D, T476X2S, T477X2A, T477X2D, T477X2S, T478X2A, T478X2D, T478X2S, T4792XA, T4792XD, T4792XS, T480X2A, T480X2D, T480X2S, T481X2A, T481X2D, T481X2S, T48202A, T48202D, T48202S, T48292A, T48292D, T48292S, T483X2A, T483X2D, T483X2S, T484X2A, T484X2D, T484X2S, T485X2A, T485X2D, T485X2S, T486X2A, T486X2D, T486X2S, T48902A, T48902D, T48902S, T48992A, T48992D, T48992S, T490X2A, T490X2D, T490X2S, T491X2A, T491X2D, T491X2S, T492X2A, T492X2D, T492X2S, T493X2A, T493X2D, T493X2S, T494X2A, T494X2D, T494X2S, T495X2A, T495X2D, T495X2S, T496X2A, T496X2D, T496X2S, T497X2A, T497X2D, T497X2S, T498X2A, T498X2D, T498X2S, T4992XA, T4992XD, T4992XS, T500X2A, T500X2D, T500X2S, T501X2A, T501X2D, T501X2S, T502X2A, T502X2D, T502X2S, T503X2A T503X2D, T503X2S, T504X2A, T504X2D, T504X2S, T505X2A, T505X2D, T505X2S, T506X2A, T506X2D, T506X2S, T507X2A, T507X2D, T507X2S, T508X2A, T508X2D, T508X2S, T50902A, T50902D, T50902S, T50992A, T50992D, T50992S, T50A12A, T50A12D, T50A12S, T50A22A, T50A22D, T50A22S, T50A92A, T50A92D, T50A92S, T50B12A, T50B12D, T50B12S, T50B92A, T50B92D, T50B92S, T50Z12A, T50Z12D, T50Z12S, T50Z92A, T50Z92D, T50Z92S, T510X2A, T510X2D, T510X2S, T511X2A, T511X2D, T511X2S, T512X2A, T512X2D, T512X2S, T513X2A, T513X2D, T513X2S, T518X2A, T518X2D, T518X2S, T5192XA, T5192XD, T5192XS, T520X2A, T520X2D, T520X2S, T521X2A, T521X2D, T521X2S, T522X2A, T522X2D, T522X2S, T523X2A, T523X2D, T523X2S, T524X2A, T524X2D, T524X2S, T528X2A, T528X2D, T528X2S, T5292XA, T5292XD, T5292XS, T530X2A, T530X2D, T530X2S, T531X2A, T531X2D, T531X2S, T532X2A, T532X2D, T532X2S, T533X2A, T533X2D, T533X2S, T534X2A, T534X2D, T534X2S, T535X2A, T535X2D, T535X2S, T536X2A, T536X2D, T536X2S, T537X2A,

	T537X2D, T537X2S, T5392XA, T5392XD, T5392XS, T540X2A, T540X2D, T540X2S, T541X2A, T541X2D, T541X2S, T542X2A, T542X2D, T542X2S, T543X2A, T543X2D, T543X2S,T5492XA, T5492XD, T5492XS, T550X2A, T550X2D, T550X2S, T551X2A, T551X2D, T551X2S, T560X2A, T560X2D, T560X2S,T561X2A, T561X2D, T561X2S, T562X2A, T562X2D, T562X2S, T563X2A, T563X2D, T563X2S, T564X2A, T564X2D, T564X2S, T565X2A, T565X2D, T565X2S, T566X2A, T566X2D, T566X2S, T567X2A, T567X2D, T567X2S, T56812A, T56812D, T56812S,T56892A, T56892D, T56892S, T5692XA, T5692XD, T5692XS, T570X2A, T570X2D, T570X2S, T571X2A, T571X2D, T571X2S, T572X2A, T572X2D, T572X2S, T573X2A, T573X2D, T573X2S, T578X2A, T578X2D, T578X2S, T5792XA, T5792XD, T5792XS, T5802XA, T5802XD, T5802XS, T5812XA,T5812XD, T5812XS, T582X2A, T582X2D, T582X2S, T588X2A, T588X2D, T588X2S, T5892XA, T5892XD, T5892XS, T590X2A, T590X2D, T590X2S, T591X2A, T591X2D, T591X2S, T592X2A, T592X2D, T592X2S, T593X2A, T593X2D, T593X2S, T594X2A, T594X2D, T594X2S, T595X2A, T595X2D, T595X2S, T596X2A, T596X2D, T596X2S, T597X2A, T597X2D, T597X2S, T59812A, T59812D, T59812S, T59892A, T59892D, T59892S, T5992XA,T5992XD, T5992XS, T600X2A, T600X2D, T600X2S, T601X2A, T601X2D,T601X2S, T602X2A, T602X2D, T602X2S, T603X2A, T603X2D, T603X2S, T604X2A, T604X2D, T604X2S, T608X2A, T608X2D, T608X2S, T6092XA, T6092XD, T6092XS, T6102XA, T6102XD, T6102XS, T6112XA, T6112XD, T6112XS, T61772A, T61772D, T61772S,T61782A,T61782D, T61782S, T618X2A, T618X2D, T618X2S, T6192XA, T6192XD, T6192XS, T620X2A, T620X2D, T620X2S, T621X2A, T621X2D, T621X2S, T622X2A, T622X2D, T622X2S, T628X2A, T628X2D, T628X2S, T6292XA, T6292XD, T6292XS, T63002A, T63002D, T63002S, T63012A, T63012D, T63012S, T63022A, T63022D, T63022S, T63032A, T63032D, T63032S, T63042A, T63042D, T63042S, T63062A, T63062D, T63062S, T63072A, T63072D, T63072S,T63082A, T63082D, T63082S, T63092A, T63092D, T63092S, T63112A, T63112D, T63112S, T63122A, T63122D, T63122S, T63192A, T63192D, T63192S, T632X2A, T632X2D, T632X2S, T63302A, T63302D, T63302S, T63312A, T63312D, T63312S, T63322A, T63322D, T63322S, T63332A, T63332D, T63332S, T63392A, T63392D, T63392S, T63412A, T63412D, T63412S, T63422A, T63422D, T63422S, T63432A, T63432D, T63432S, T63442A, T63442D, T63442S, T63452A, T63452D, T63452S, T63462A, T63462D, T63462S, T63482A, T63482D, T63482S, T63512A, T63512D, T63512S, T63592A, T63592D, T63592S, T63612A, T63612D, T63612S, T63622A, T63622D, T63622S, T63632A, T63632D, T63632S, T63692A, T63692D, T63692S, T63712A, T63712D, T63712S, T63792A, T63792D, T63792S, T63812A, T63812D, T63812S, T63822A, T63822D, T63822S, T63832A, T63832D, T63832S, T63892A, T63892D, T63892S, T6392XA, T6392XD, T6392XS, T6402XA, T6402XD, T6402XS, T6482XA, T6482XD, T6482XS, T650X2A, T650X2D, T650X2S, T651X2A, T651X2D, T651X2S, T65212A, T65212D, T65212S, T65222A, T65222D, T65222S, T65292A, T65292D, T65292S, T653X2A, T653X2D, T653X2S, T654X2A, T654X2D, T654X2S, T655X2A, T655X2D, T655X2S, T656X2A, T656X2D, T656X2S, T65812A, T65812D, T65812S, T65822A, T65822D, T65822S, T65832A, T65832D, T65832S, T65892A, T65892D, T65892S, T6592XA, T6592XD, T6592XS, T71112A, T71112D, T71112S, T71122A, T71122D, T71122S, T71132A, T71132D, T71132S, T71152A, T71152D, T71152S, T71162A, T71162D, T71162S, T71192A, T71192D, T71192S, T71222A, T71222D, T71222S, T71232A, T71232D, T71232S, X710XXA, X710XXD, X710XXS, X711XXA, X711XXD, X711XXS, X712XXA, X712XXD, X712XXS, X713XXA, X713XXD, X713XXS, X718XXA, X718XXD, X718XXS, X719XXA, X719XXD, X719XXS, X72XXXA, X72XXXD, X72XXXS, X730XXA, X730XXD, X730XXS, X731XXA, X731XXD, X731XXS, X732XXA, X732XXD, X732XXS, X738XXA, X738XXD, X738XXS, X739XXA, X739XXD, X739XXS, X7401XA, X7401XD, X7401XS, X7402XA, X7402XD, X7402XS, X7409XA, X7409XD, X7409XS, X748XXA, X748XXD, X748XXS, X749XXA, X749XXD, X749XXS, X75XXXA, X75XXXD, X75XXXS, X76XXXA, X76XXXD, X76XXXS, X770XXA, X770XXD, X770XXS, X771XXA, X771XXD, X771XXS, X772XXA, X772XXD, X772XXS, X773XXA, X773XXD, X773XXS, X778XXA, X778XXD, X778XXS, X779XXA, X779XXD, X779XXS, X780XXA, X780XXD, X780XXS, X781XXA, X781XXD, X781XXS, X782XXA, X782XXD, X782XXS, X788XXA, X788XXD, X788XXS,

	X789XXA, X789XXD, X789XXS, X79XXXA, X79XXXD, X79XXXS, X80XXXA, X80XXXD, X80XXXS, X810XXA, X810XXD, X810XXS, X811XXA, X811XXD, X811XXS, X818XXA, X818XXD, X818XXS, X820XXA, X820XXD, X820XXS, X821XXA, X821XXD, X821XXS, X822XXA, X822XXD, X822XXS, X828XXA, X828XXD, X828XXS, X830XXA, X830XXD, X830XXS, X831XXA, X831XXD, X831XXS, X832XXA, X832XXD, X832XXS, X838XXA, X838XXD, X838XXS, Z915
Screening and history of mental health and substance abuse codes	R780, R781, R782, R783, R784, R785, R786, Z046, Z134, Z72810, Z72811, Z8651, Z8659, Z87891, Z91410, Z91411, Z91412, Z91419, Z9149
Miscellaneous mental health disorders	F061, F068, F440, F441, F442, F444, F445, F446, F447, F4481, F4489, F449, F450, F451, F4520, F4521, F4522, F4529, F4541, F4542, F458, F459, F481, F5000, F5001, F5002, F502, F508, F5081, F5082, F5089, F509, F5101, F5102, F5103, F5104, F5105, F5109, F5111, F5112, F5113, F5119, F513, F514, F515, F518, F519, F520, F521, F5221, F5222, F5231, F5232, F524, F525, F526, F528, F529, F53, F54, F59, F640, F641, F648, F649, F650, F651, F652, F653, F654, F6550, F6551, F6552, F6581, F6589, F659, F66, F6810, F6811, F6812, F6813, F688, F99, O906, R37, R4589, Z87890, Z9183
Alcohol related disorders	F1010, F10120, F10121, F10129, F1014, F10150, F10151, F10159, F10180, F10181, F10182, F10188, F1019, F1020, F1021, F10220, F10221, F10229, F10230, F10231, F10232, F10239, F1024, F10250, F10251, F10259, F1026, F1027, F10280, F10281, F10282, F10288, F1029, F10920, F10921, F10929, F1094, F10950, F10951, F10959, F1096, F1097, F10980, F10981, F10982, F10988, F1099, G621, I426, K2920, K2921, K700, K7010,K7011, K702, K7030, K7031, K7040, K709, K7041, K852, K860, E244, G312, G721
Pregnancy related alcohol disorders	O99310, O99311, O99312, O99313, O99314, O99315, O355XX0, O355XX1, O355XX2, O355XX3, O355XX4, O355XX5, O355XX9, O99320, O99321, O99322, O99323, O99324, O99325
Opioid related disorders	F1110, F11120, F11121, F11122, F11129, F1114, F11150, F11151, F11159, F11181, F11182, F11188, F1119, F1120, F1121, F11220, F11221, F11222, F11229, F1123, F1124, F11250, F11251, F11259, F11281, F11282, F11288, F1129, F1190, F11920, F11921, F11922, F11929, F1193, F1194, F11950, F11951, F11959, F11981, F11982, F11988, F1199
Cannabis related disorders	F1210, F12120, F12121, F12122, F12129, F12150, F12151, F12159, F12180, F12188, F1219, F1220, F1221, F12220, F12221, F12222, F12229, F12250, F12251, F12259, F12280, F12288, F1229, F1290, F12920, F12921, F12922, F12929, F12950, F12951, F12959, F12980, F12988, F1299
Sedative, hypnotic, or anxiolytic related disorders	F1310, F13120, F13121, F13129, F1314, F13150, F13151, F13159, F13180, F13181,F13182, F13188, F1319, F1320, F1321, F13220, F13221, F13229, F13230, F13231, F13232, F13239, F1324, F13250, F13251, F13259, F1326, F1327, F13280, F13281, F13282, F13288, F1329, F1390, F13920, F13921, F13929, F13930, F13931, F13932, F13939, F1394, F13950, F13951, F13959, F1396, F1397, F13980, F13981, F13982, F13988, F1399
Cocaine related disorders	F1410, F14120, F14121, F14122, F14129, F1414, F14150, F14151, F14159, F14180, F14181, F14182, F14188, F1419, F1420, F1421, F14220, F14221, F14222, F14229, F1423, F1424, F14250, F14251, F14259, F14280, F14281, F14282, F14288, F1429, F1490, F14920, F14921, F14922, F14929, F1494, F14950, F14951, F14959, F14980, F14981, F14982, F14988, F1499
Other stimulant related disorders	F1510, F15120, F15121, F15122, F15129, F1514, F15150, F15151, F15159, F15180, F15181, F15182, F15188, F1519, F1520, F1521, F15220, F15221, F15222, F15229, F1523, F1524, F15250, F15251, F15259, F15280, F15281, F15282, F15288, F1529, F1590, F15920, F15921, F15922, F15929, F1593, F1594, F15950, F15951, F15959, F15980, F15981, F15982, F15988, F1599
Hallucinogen related disorders	F1610, F16120, F16121, F16122, F16129, F1614, F16150, F16151, F16159, F16180, F16183, F16188, F1619, F1620, F1621, F16220, F16221, F16229, F1624, F16250, F16251, F16259, F16280, F16283, F16288, F1629, F1690, F16920, F16921, F16929, F1694, F16950, F16951, F16959, F16980, F16983, F16988, F1699
Inhalant related disorders	F1810, F18120, F18121, F18129, F1814, F18150, F18151, F18159, F1817, F18180, F18188, F1819, F1820, F1821, F18220, F18221, F18229, F1824, F18250, F18251, F18259, F1827, F18280, F18288, F1829, F1890, F18920, F18921, F18929, F1894, F18950, F18951, F18959, F1897, F18980, F18988, F1899

Other psychoactive substance related disorders	F1910, F19120, F19121, F19122, F19129, F1914, F19150, F19151, F19159, F1916, F1917, F19180, F19181, F19182, F19188, F1919, F1920, F1921, F19220, F19221, F19222, F19229, F19230, F19231, F19232, F19239, F1924, F19250, F19251, F19259, F1926, F1927, F19280, F19281, F19282, F19288, F1929, F1990, F19920, F19921, F19922, F19929, F19930, F19931, F19932, F19939, F1994, F19950, F19951, F19959, F1996, F1997, F19980, F19981, F19982, F19988, F1999
Abuse	F550, F551, F552, F553, F554, F558
Poisoning	T400X1A, T400X1D, T400X1S, T400X3A, T400X3D, T400X3S, T400X4A, T400X4D, T400X4S, T400X5A, T400X5D, T400X5S, T401X1A, T401X1D, T401X1S, T401X3A, T401X3D, T401X3S, T401X4A, T401X4D, T401X4S, T401X5A, T401X5D, T401X5S, T402X1A, T402X1D, T402X1S, T402X3A, T402X3D, T402X3S, T402X4A, T402X4D, T402X4S, T402X5A, T402X5D, T402X5S, T403X1A, T403X1D, T403X1S, T403X3A, T403X3D, T403X3S, T403X4A, T403X4D, T403X4S, T403X5A, T403X5D, T403X5S, T404X1A, T404X1D, T404X1S, T404X3A, T404X3D, T404X3S, T404X4A, T404X4D, T404X4S, T404X5A, T404X5D, T404X5S, T405X1A, T405X1D, T405X1S, T405X3A, T405X3D, T405X3S, T405X4A, T405X4D, T405X4S, T405X5A, T405X5D, T405X5S, T407X1A, T407X1D, T407X1S, T407X3A, T407X3D, T407X3S, T407X4A, T407X4D, T407X4S, T407X5A, T407X5D, T407X5S, T408X1A, T408X1D, T408X1S, T408X3A, T408X3D, T408X3S, T408X4A, T408X4D, T408X4S, T408X5A, T408X5D, T408X5S, T40901A, T40901D, T40901S, T40903A, T40903D, T40903S, T40904A, T40904D, T40904S, T40905A, T40905D, T40905S, T40991A, T40991D, T40991S, T40993A, T40993D, T40993S, T40994A, T40994D, T40994S, T40995A, T40995D, T40995S
Infancy related disorders	P043, P0441, P0449, P961, P962, Q860
New codes from Medical Care	T40605A, T40605D, T40605S, T40695A, T40695D, T40695S, T40601A, T40601D, T40601S, T40604A, T40604D, T40604S, T40691A, T40691D, T40691S, T40694A, T40694D, T40694S
Opioid use disorders	F1110, F11120, F11121, F11122, F11129, F1114, F11150, F11151, F11159, F11181, F11182, F11188, F1119, F1120, F1121, F11220, F11221, F11222, F11229, F1123, F1124, F11250, F11251, F11259, F11281, F11282, F11288, F1129, F1190, F11920, F11921, F11922, F11929, F1193, F1194, F11950, F11951, F11959, F11981, F11982, F11988, F1199, T400X1A, T400X1D, T400X1S, T400X3A, T400X3D, T400X3S, T400X4A, T400X4D, T400X4S, T400X5A, T400X5D, T400X5S, T402X1A, T402X1D, T402X1S, T402X3A, T402X3D, T402X3S, T402X4A, T402X4D, T402X4S, T402X5A, T402X5D, T402X5S, T40605A, T40605D, T40605S, T40695A, T40695D, T40695S, T40601A, T40601D, T40601S, T40604A, T40604D, T40604S, T40691A, T40691D, T40691S, T40694A, T40694D, T40694S

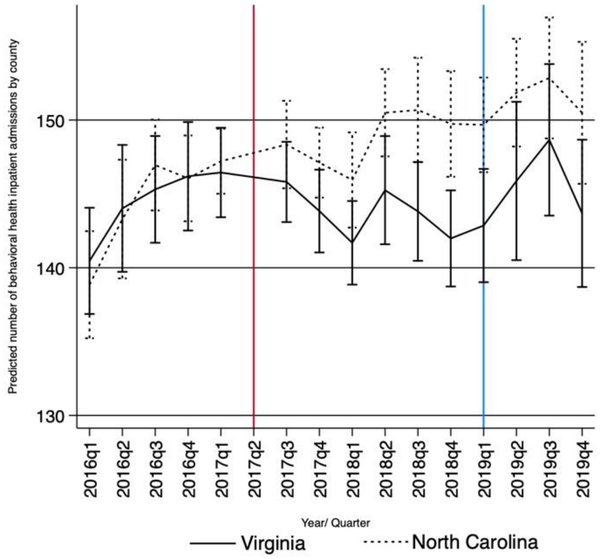

Figure S1. Impact of adoption of ARTS [a] and Medicaid Expansion Predicted number of mental illness inpatient admissions by county/quarter. The red vertical line indicates the first quarter of the ARTS [a] program, and the blue vertical line indicates the first quarter of Medicaid expansion in Virginia. [a] Addiction and Recovery Treatment Services.

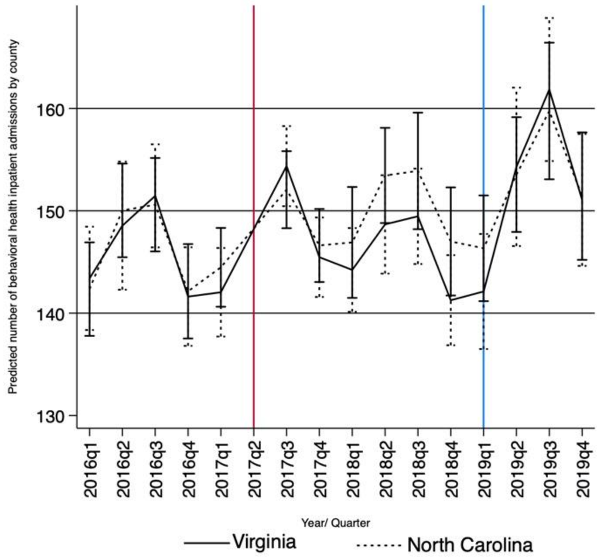

Figure S2. Impact of adoption of ARTS [a] and Medicaid Expansion Predicted number of SUD inpatient admissions by county/quarter. The red vertical line indicates the first quarter of the ARTS [a] program, and the blue vertical line indicates the first quarter of Medicaid expansion in Virginia. [a] Addiction and Recovery Treatment Services.
